# *GLA* Mutations Suppress Autophagy and Stimulate Lysosome Generation in Fabry Disease

**DOI:** 10.3390/cells13050437

**Published:** 2024-03-01

**Authors:** Ping Li, Yuqian Xi, Yanping Zhang, Abdus Samad, Wenli Lan, Ya Wu, Jiayu Zhao, Guangxin Chen, Changxin Wu, Qiuhong Xiong

**Affiliations:** 1 Institutes of Biomedical Sciences, The Key Laboratory of Chemical Biology and Molecular Engineering of Ministry of Education of China, The Key Laboratory of Medical Molecular Cell Biology of Shanxi Province, Shanxi University, Taiyuan 030006, China; 202123105013@email.sxu.edu.cn (Y.X.); 201923105006@email.sxu.edu.cn (Y.Z.); 202123105006@email.sxu.edu.cn (W.L.); 202223002019@email.sxu.edu.cn (Y.W.); 202223105017@email.sxu.edu.cn (J.Z.); chengx@sxu.edu.cn (G.C.); cxw20@sxu.edu.cn (C.W.); 2 Department of Biochemistry, Abdul Wali Khan University Mardan, Mardan 23200, Pakistan; sabdus591@gmail.com

**Keywords:** fabry disease, kidney, GLA, autophagy, lysosome, mTOR, LAMP2

## Abstract

Fabry disease (FD) is an X-linked recessive inheritance lysosomal storage disorder caused by pathogenic mutations in the *GLA* gene leading to a deficiency of the enzyme alpha-galactosidase A (α-Gal A). Multiple organ systems are implicated in FD, most notably the kidney, heart, and central nervous system. In our previous study, we identified four *GLA* mutations from four independent Fabry disease families with kidney disease or neuropathic pain: c.119C>A (p.P40H), c.280T>C (C94R), c.680G>C (p.R227P) and c.801+1G>A (p.L268fsX3). To reveal the molecular mechanism underlying the predisposition to Fabry disease caused by *GLA* mutations, we analyzed the effects of these four *GLA* mutations on the protein structure of α-galactosidase A using bioinformatics methods. The results showed that these mutations have a significant impact on the internal dynamics and structures of GLA, and all these altered amino acids are close to the enzyme activity center and lead to significantly reduced enzyme activity. Furthermore, these mutations led to the accumulation of autophagosomes and impairment of autophagy in the cells, which may in turn negatively regulate autophagy by slightly increasing the phosphorylation of mTOR. Moreover, the overexpression of these GLA mutants promoted the expression of lysosome-associated membrane protein 2 (LAMP2), resulting in an increased number of lysosomes. Our study reveals the pathogenesis of these four *GLA* mutations in FD and provides a scientific foundation for accurate diagnosis and precise medical intervention for FD.

## 1. Introduction

Fabry disease (FD; OMIM 301500) is a lysosomal storage disease caused by a deficiency of α-galactosidase A that was first reported by two dermatologists, William Anderson (England) and Johannes Fabry (Germany) in 1898 [1]. Patients with FD have various symptoms and most of them have a single symptom in the early stage of onset. Some clinical symptoms, such as gastrointestinal symptoms (nausea, diarrhea, abdominal pain), neuropathic pain, angiokeratoma, hypohidrosis, acroparesthesia, and corneal opacities (cornea verticillata) appear in childhood or adolescence [2]. Along with the gradual increase in onset time, in adulthood, most patients begin to suffer from multiorgan injury, including kidney disease and cardiac and central nervous system injury, which contributes to morbidity and early mortality, with a mean survival rate of approximately 55 years for males and 70 years for females [3]. Multiple atypical variants predominantly involve one organ, with significant residual enzyme activity. The most frequently targeted organs include the kidneys [4,5], heart [6,7], peripheral nerves, and skin. Diagnosis of these variants is difficult, as there is no clear correlation between genotype and phenotype [8].

The α-galactosidase A, with 429 amino acids, is encoded by the *GLA* gene (located at position Xq22.1) and contains seven exons and six introns [9,10]. GLA functions as a homodimer, and each monomer contains an enzyme activity center [11]. α-Galactosidase A is present in lysosomes and is distributed throughout the body to catalyze the intracellular hydrolysis of terminal nonreducing α-D-galactose residues in α-D-galactosides, including galactooligosaccharides, galactomannans, and galactolipids. Fabry disease is caused by the loss of α-galactosidase A activity, which prevents cells from degrading glycosphingolipids with terminal D-galactosyl residues, especially spherical triacylceramides (Gb3). These substrates accumulate in almost all organs with age and gradually cause progressive multisystem disease [12].

Autophagy is a way for cells to maintain their own dynamic balance. The process of autophagy includes five main stages: the generation of the autophagosome membrane, the formation of autophagic vesicles through the extension of the bilayer membrane, the maturation of autophagosomes, fusion with lysosomes, and degradation of engulfed cargoes [13]. The initiation of autophagy is regulated by mTOR, whose phosphorylation leads to enhanced activity, thus inhibiting the initiation of autophagy [14]. In Fabry disease, *GLA* gene mutations cause impaired α-galactosidase A activity leading to the ineffective degradation of lipid substrates in cells, resulting in massive accumulation [15,16]. The negative feedback regulation of autophagy by a large amount of accumulated lipid substrates leads to an imbalance in intracellular homeostasis [17].

In this study, we focused on the four *GLA* mutations identified in four independent FD families with kidney disease or neuropathic pain in our laboratory previously [18,19], and the effects of the different mutations on protein structure were analyzed *in silico*. Cells stably overexpressing *GLA* wild-type or mutants were constructed and the effects of these *GLA* mutations on autophagy and lysosomes were analyzed. The aim of this study was to elucidate the potential molecular mechanism of the *GLA* mutations resulting in FD.

## 2. Materials and Methods

### 2.1. Evolutionary Conservation and Structure Preparation

The evolutionary conservation of amino acid residue alterations was analyzed by comparison across different species using the Megalign tool from DNAStar. The crystal structure of the native GLA protein with the PDB accession code 3S5Z was retrieved from the protein database [20]. Prior to MD simulations, the protein structure was prepared and the missing loops were built by the loop modeling module of Chimera [21]. Afterward with the use of MMFF94S in the molecular operating environment (MOE), energy minimization was performed [22]. Since the mutant structures of GLA have not yet been solved, we employed PyMOL to introduce mutations at a particular location [23]. The dynamic and structural changes in all five systems, including the GLA wild type and mutants (GLA-P40H, GLA-C94R, GLA-R227P, and GLA-L268fsX3) were examined and compared using 200 ns of MD simulations.

### 2.2. Molecular Dynamic Simulation

To examine the conformational alterations and stability of native GLA and its mutant versions, 200 ns of molecular dynamic (MD) simulations were performed using AMBER 20 software with the ff14SB force field [24]. The systems were solvated by the tip3p water model with the following box dimensions: X = 56.643, Y = 72.313, and Z = 67.852. In addition to neutralizing the systems, counterions were added and the systems were subjected to energy minimizations; for instance, six thousand steps were used for steepest descent and three thousand steps were used for conjugate gradient energy minimization [25]. The systems were gradually warmed to approximately 300 K for up to 200 ps. To achieve equilibrium for each system, the system density was initially brought into equilibrium with weak restrictions for 2 ns later, the entire system was brought into equilibrium under a constant pressure for 2 ns. A 100-ns MD rung was applied to the systems after they reached equilibrium, while a Langevin thermostat was used to regulate the temperature (1 atm, 300 K). The Particle Mesh Ewald (PME) algorithm was applied to calculate long-range electrostatic interactions. The long-range electrostatic and van der Waals interactions were both set at 10.0 Å for cutoff distances, while for covalent bonds involving hydrogen atoms, the SHAKE algorithm was used [26]. All MD steps for each system were carried out by utilizing the accelerated GPU commands of PMEMD.CUDA. For post-MD simulation analysis, the cpptraj module of Amber 2021 was utilized; for graphical representation, OriginPro 2021 was used [27].

### 2.3. Dynamic Cross-Correlation Analysis (DCCM)

DCCM is a valuable method for employing molecular dynamic simulations to unravel the intricate correlation patterns among residues within a three-dimensional matrix representation of biological systems [28]. The following equation was used for computing the cross correlations:

Cij=∆ri.∆rj∆ri2∆rj21/2

where the variables ∆ri and ∆rj indicate the mean positions of the ith and jth atoms, respectively. where the average time of all the trajectories generated by MD simulations is measured using the angular brackets. A positive *Cij* value indicates correlated movement, such as movement in the same direction, while a negative *Cij* value indicates significant anticorrelation movements between the residues. For the DCCM analysis, Cpptraj was used, while Origin 2021 was used for graphical representations.

### 2.4. Principal Component Analysis (PCA)

We captured high-amplitude principal motions of proteins by using the cpptraj package of Amber tool version 2021 for principal component analysis (PCA) [29]. For the whole trajectory, the covariance matrix was created by using C Cartesian coordinates of 5000 snapshots for each system to examine the dynamic behavior of each system. The matrix displays the dynamic behavior of atoms, which is based on the average position of the alpha carbon atoms. The diagonalized position of the covariance matrix shows eigenvalues and eigenvectors; the eigenvectors show the direction of high-amplitude motion, while the eigenvalues show the mean square fluctuations. To track the moments for each system, PC1 and PC2 were calculated and plotted.

### 2.5. Cell Culture and Ctable Cell Line Construction

HEK293T cells obtained from Haixing Bioscience (Suzhou, China) were maintained in DMEM supplemented with 10% (*v*/*v*) FBS (Sorfa, Beijing, China), 100 U/mL penicillin, and 100 mg/mL streptomycin at 37 °C and 5% CO_2_. We used a plasmid harboring cDNA sequences encoding human *GLA* full length (pEGFP-GLA) [18] to produce the C-terminus Flag-tagged GLA wildtype lentivirus plasmid and four GLA mutants, c.119C>A (p.P40H), c.280T>C (C94R), c.680G>C (p.R227P), and c.801+1G>A (p.L268fsX3) were subsequently generated by site-directed mutagenesis. The main plasmid of the lentivirus containing the *GLA* wildtype or mutant cDNA was mixed with psPAX2 and pMD2.G at a mass ratio of 3:2:1 and then transfected with PEI at a ratio of 3:1. The cell culture supernatant (containing virus particles) was collected on the second and third days after transfection, and the supernatant was subsequently sterilized by filtration through a 0.45 μm filter after centrifugation and stored at −80 °C. A total of 600,000 cells were seeded in each 30 mm Petri dish, the culture medium and the collected virus supernatant were mixed at a 2:1 ratio, and the cultured cells were continuously infected for three days. Later, puromycin (1–10 μg/mL) was added to the fresh medium for stable cell line screening.

### 2.6. GLA Enzyme Activity Assay

HEK293T cells were cultured and transfected with plasmids containing either *GLA* wildtype or mutant. At 48 h post-transfection, the cells were collected after centrifugation at 1500 rpm using a benchtop centrifuge. Every 5 million cells were resuspended in 1 mL of extraction buffer and sonicated. The lysates were cleared by centrifugation at 15,000× *g* for 10 min at 4 °C. The GLA enzyme activity in the supernatants was measured according to the manufacturer’s instructions (Solarbio, Beijing, China, Cat. No. BC2575).

In detail, the samples were seeded in a 96-well plate, and the assay reagents were added and mixed thoroughly according to the instructions provided by the supplier. The absorbance A at 400 nm was measured, and the ΔA = A measurement-A control was calculated. For the calculation of α-GAL activity, a standard curve was established based on the absorbance (x) and concentration (y, nmol/mL) of the standard sample, and ΔA was taken as the standard curve to calculate the amount of product (nmol/mL) produced by the samples. For the definition of enzyme activity, 1 nmol of p-nitrophenol per hour per 10,000 cells was defined as an enzyme activity unit, and α-GAL activity (nmol/h/104 cell) was calculated as (y × V1) ÷ (500 × V ÷ V 2) ÷T = 0.028 × y. (V1: total volume of the reaction system, 0.07 mL; V: sample volume used in the reaction system, 0.01 mL; V2: volume of added extract solution, 1 mL; 500: total number of cells, 5 million; T: reaction time, 0.5 h)

### 2.7. Western Blot Analysis

Stable overexpression cells were grown to 90% confluence in 6-well plates and lysed in RIPA buffer supplemented with 1 mM DTT, 1 mM benzamidine, 1 mM PMSF, and 1× protease inhibitor cocktail. The cell suspensions were incubated for 15 min on ice, followed by short sonication. The supernatants of the lysates were collected after centrifugation at 10,000 rpm for 10 min at 4 °C. The samples were heated with 1× SDS loading buffer at 98 °C for 5 to 10 min. Following 10% or 15% SDS–PAGE and wet blot analysis, the blots were probed with one of the following primary antibodies in TBST: anti-p-mTOR (phospho-S2448) (Bioworld, Nanjing, China, Cat. No. BS4706), anti-mTOR (S2442) (Bioworld, Cat. No. BS3611), anti-LC3 (Abcam, Cambridge, UK, Cat. No. ab51520), anti-p62 (Abcam, Cat. No. ab56416), anti-LAMP2 (Proteintech, Wuhan, China, Cat. No. 66301-1-Ig), or anti-GAPDH (Proteintech, Cat. No. 60004-1-Ig). The blots were incubated with horseradish peroxidase-conjugated secondary antibody after three washes, and the signals were visualized using a chemiluminescence (ECL) system (Engreen, Beijing, China, Cat. No. 29100).

### 2.8. Immunofluorescence Analysis

For live-cell imaging, HEK293T cells overexpressing *GLA* wildtype or mutants were pre-transfected with pCS2-mCherry-LC3 or pCS2-mCherry-GFP-LC3 plasmid for 24 h and subsequently transferred to a μ-Dish (Φ 35 mm, NEST). For pCS2-mCherry-LC3 transfection, after 12 h of cell attachment, the cells were washed once with PBS, and 2 mL of serum-free medium supplemented with 50 nM LysoTracker Green (Beyotime, Shanghai, China, C1047S), a green fluorescent probe that can be selectively retained in lysosomes, was added. After coincubation for 30 min in the cell incubator, the medium was replaced with fresh medium without LysoTracker Green to avoid nonspecific labeling caused by an excessively long staining time. Confocal images of the cells were taken immediately with a laser scanning microscope (Zeiss, Oberkochen, Germany). The flux of autophagy in cells can be measured by the colocalization of LC3 with lysosomes. The excitation wavelength of LysoTracker Green was 488 nm, and the emission wavelength was 500–550 nm. mCherry-LC3 was excited at 561 nm and emitted from 570 to 620 nm. Images were processed using ImageJ 1.52a.

### 2.9. Statistical Analysis

All the data were presented as mean ± SD from at least three independent experiments. Statistical analysis was performed using GraphPad Prism software version 8. Multiple group comparisons were conducted using one-way ANOVA followed by the least significant difference test (* *p* < 0.05, ** *p* < 0.01, *** *p* < 0.001).

## 3. Results

### 3.1. Abnormal Three-Dimensional Structure of α-Galactosidase A Caused by GLA Mutations

**Figure 1:**
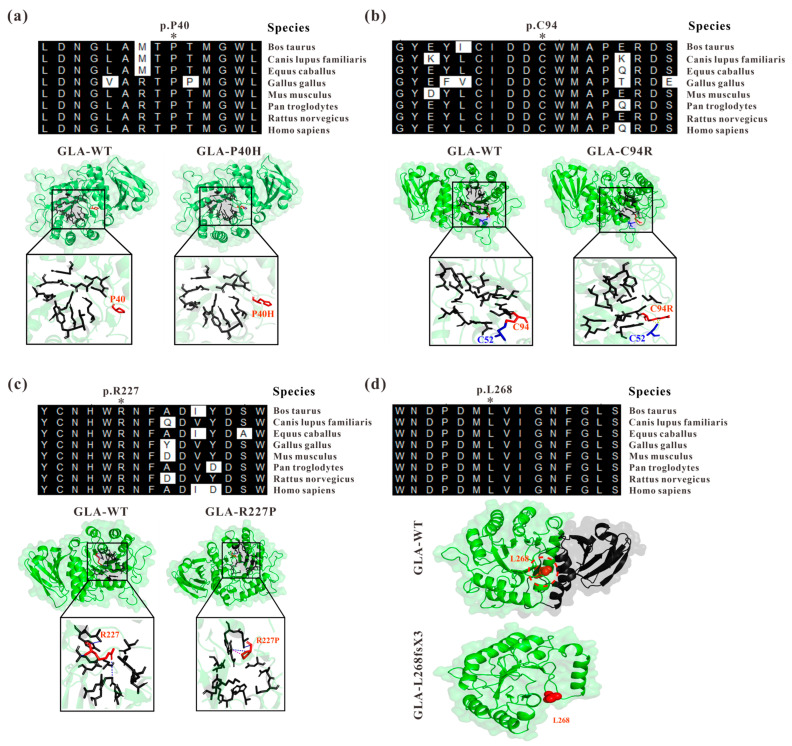
Analysis of GLA mutants by evolutionary conservation of altered amino acids and protein structures modelled by mutagenesis module of PyMOL compared to those of the wild type. (**a**) GLA-P40H; (**b**) GLA-C94R; (**c**) GLA-R227P; (**d**) GLA-L268fsX3. The NCBI accession numbers are as follows: *Bos Taurus*: NP_001179665; *Canis lupus familiaris*: XP_538109; *Equus caballus*: XP_001492699; *Gallus gallus*: XP_420183; *Mus musculus*: NP_038491; *Pantroglodytes*: XP_003954083; *Rattus norvegicus*: NP_001102290; and *Homo sapiens*: NP_000160.

In a previous study, we identified four *GLA* mutations from four independent Fabry disease families, *GLA* c.119C>A (p.P40H), c.280T>C (p.C94R), c.680G>C (R227P), and the splicing mutation c.801+1G>A [18,19]. Through sequence alignment, we found that P40, C94, R227, and L268 are highly conserved among different species (Figure 1). Based on the crystal structure of the GLA wildtype protein with the PDB accession code 3S5Z [20], the structures of the mutated proteins were modelled by PyMOL to introduce mutations at a particular location (Figure 1). For *GLA* c.119C>A, the amino acid at position 40 of the GLA protein was mutated from proline (P) to histidine (H) (Figure 1a). Although histidine and proline are both α-amino acid structures, histidine has one more amino group than proline, resulting in it being unembedded in the GLA tertiary structure [30]. This might lead to the loose structure of the GLA enzyme activity region and the reduced binding to the substrate, resulting in the accumulation of GB3 and the onset of disease. The cysteine at position 94 (Cys94 or C94) is highly conserved across different species, and the *GLA* c.280T>C mutation disrupted the disulfide bond originally formed between C94 and C52 in GLA (Figure 1b). The mature GLA protein monomers have 12 cysteine residues at positions 52, 56, 63, 90, 94, 142, 172, 174, 202, 223, 378, and 382 in the polypeptide chain. In addition to the unpaired C90 and C174 residues, the other 10 cysteine residues were pairwise paired to form five disulfide bonds, namely, C52-C94, C56-C63, C142-C172, C202-C223, and C378-C382. The presence of disulfide bonds promotes the gathering of amino acid residues in different spatial regions of proteins, and the peptide chain is folded to form a relatively stable spatial topology, which plays a decisive role in the correct folding of proteins and the maintenance of structural stability [11,31]. R227 is one of the key amino acids forming the active site of α-galactosidase A including W47, D92, D93, Y134, C142, K168, D170, E203, L206, Y207, D231, D266, and M267, with seven rings from the first domain surrounding the enzyme active site, which is the key region for the chimeric catalytic effect of α-galactosidase A [11]. The *GLA* c.680G>C (R227P) mutation (Figure 1c) is likely to affect the enzyme activity resulting in insufficient degradation of substrates in cells for disease development. The *GLA* c.801+1G>A mutation located at the first position of *GLA* intron 5 affects *GLA* splicing resulting in a reading frameshift causing early termination of protein translation, namely, GLA p.L268IfsX3 [18]. Human GLA is a homodimer and each monomer consists of a (β/a)_8_ domain (aa 32–330) with an activity center and an antiparallel β-fold domain (aa 331–429) [11]. The c.801+1G>A mutation resulted in a truncated GLA protein, which almost completely lost the entire second domain of GLA (Figure 1d) [18].

### 3.2. Structural Perturbations upon GLA Mutations

**Figure 2:**
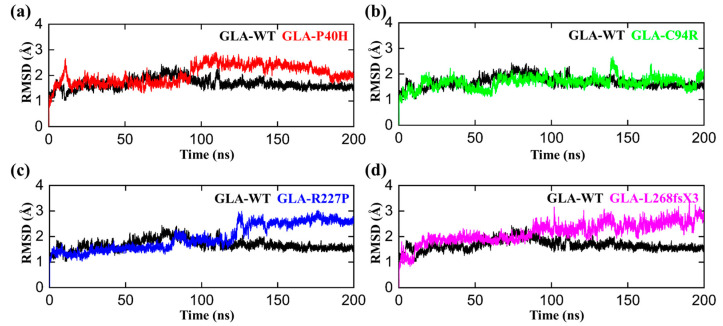
RMSD plots of the GLA wild type and mutants. The black RMSD curves in each plot represent the wild type, while the different colors indicate the GLA mutants. (**a**) GLA-P40H (red), (**b**) GLA-C94R (green), (**c**) GLA-R227P (blue), and (**d**) GLA-L268fsX3 (pink). The time in nanoseconds is presented on the *X*-axis, while the RMSD in angstroms is present on the *Y*-axis.

The conformational fluctuations of GLA after acquiring the mutations were analyzed by comparing the root mean square deviations (RMSDs) of GLA-WT, GLA-P40H, GLA-C94R, GLA-R227P, and GLA-L268fsX3 to assess the conformational stability of each mutated system [32]. The RMSD analysis demonstrated that the GLA wild type gained stability during the first 50 ns of the simulation period. Initially, up to 20 ns, slight deviations from its mean position were analyzed, but afterward, the RMSD for the wild type converged and no significant deviations in the RMSD curve remained consistent between 1 and 2 Å as shown in Figure 2. Afterwards, we analyzed the stability of the P40H mutant system in terms of RMSD. A slight increase in the RMSD curve was observed in the first and second halves of the simulation period. The P40H system attained stability at 170 ns, with an RMSD between 1.8 and 3.5 Å. Next, to reveal the dynamic stability of C95R, we calculated its stability, which indicated that the mutant system adopted seasonal stability while occasionally displaying more fluctuations at different intervals than the wild type. GLA-R227P was stable during the first 80 ns, and the RMSD of the system gradually increased to 3 Å after 120 ns. The RMSD stabilized to 150 ns. The average RMSD of this system was predicted to be 1.8 Å. GLA-L268fsX3 remained unstable until the end of the simulation. For GLA-L268fsX3, the RMSD values showed a trend that was substantially more divergent from that of the wild type, with a standard deviation of 1.3 Å. In comparison to that of the wild type, the mutant’s system showed more significant divergence from its mean position, indicating that all these mutated systems underwent structural perturbations upon mutations.

### 3.3. Flexibility Comparison of Wildtype and Mutant GLA Structures

**Figure 3:**
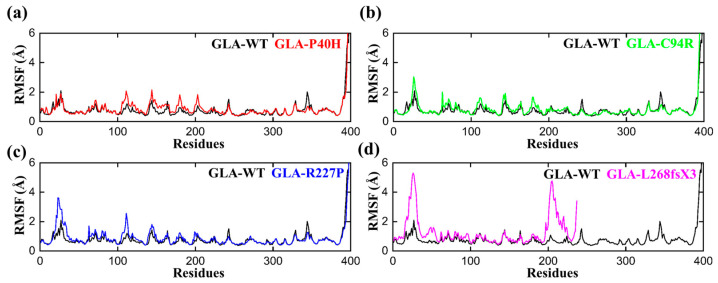
RMSF plots of the GLA wild type and mutants. The black RMSF line in each plot represents the wild type, while the different colors indicate the mutant variants of GLA. (**a**) GLA-P40H (red), (**b**) GLA-C94R (green), (**c**) GLA-R227P (blue), and (**d**) GLA-L268fsX3 (pink). The total number of residues is presented on the *X*-axis, while the value of RMSF in angstroms is presented on the *Y*-axis.

To determine the per residual flexibility, we carried out a root mean square fluctuation (RMSF) analysis to compare the per residue fluctuation patterns of the GLA-P40H, GLA-C94R, GLA-R227P, and GLA-L268fsX3 mutations to that of the wild type. There was a more consistent pattern of flexibility across all the systems, but variations could be seen in the flexibility values. Compared with that of the mutant system, the RMSF value of the wild type was lower, as shown in Figure 3. The region between 20–35 and 100–120 has greater fluctuations than any other region for all the systems. Apart from GLA-L268fsX3, which had a somewhat larger variation between 20 and 35 and between 198 and 227, the highest RMSF value was obtained for GLA-L268fsX3. These findings strongly support the finding of our previous rmsd analysis that the reported mutations had distinct effects on the internal dynamics of the residues.

### 3.4. Structural Compactness of GLA upon Acquring Mutations

**Figure 4:**
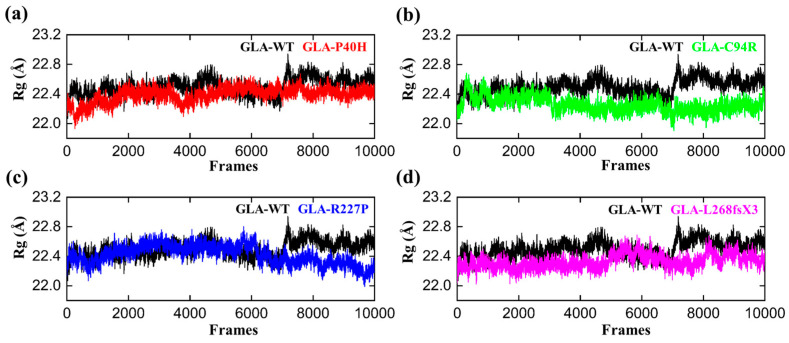
RG plots of the GLA wild type and mutants. The black RGs in each plot represent the wild type, while the different colors indicate the mutant variants of GLA. (**a**) GLA-P40H (red), (**b**) GLA-C94R (green), (**c**) GLA-R227P (blue), and (**d**) GLA-L268fsX3 (pink). The total number of frames is presented on the *X*-axis, while the value of Rg in angstroms is presented on the *Y*-axis.

We computed the radius of gyration (RG) of all the systems, including the wild type, to gain insights into how mutations affect structural compactness during molecular dynamics simulation. The reduced degree of fluctuation and the constant RG value throughout the simulation show that the system is more compact and stable. Therefore, a protein that has been folded consistently is likely to have a stable radius of gyration [33]. The average Rg value of GLA-WT was 22.3 Å. The Rg value first increased from 22.2 A to 22.4 Å up to 40 ns. Afterward, the Rg curve converged, and no significant deviation from the mean position was observed. This pattern was notably altered in the mutants (Figure 4). The Rg value of the GLA-P40H mutant was relatively greater than that of the wild type. The initial value of Rg was reported to be 22.5 Å up to 140 ns. Nevertheless, substantial convergence was observed at 150 ns, the Rg value increased from 22.5 Å to 22.7 Å, and this increase remained constant for 200 ns. Next, we analyzed the gyration radius of GLA-C94R. The Rg value increased continuously from 22.2 Å to 22.6 Å up to 20 ns, followed by a constant Rg value for GLA-C94R of up to 60 ns. Afterward, the Rg value abruptly decreased, and for the remainder of the simulation duration, the average Rg value remained at 22.4 Å. However, in the case of R227, the Rg value remained highly ambiguous; initially, the Rg curve slowly decreased up to 25 ns, followed by a gradual increase to 40 ns with an average Rg value of 22.5 Å. This value was maintained up to 120 ns; thereafter, the Rg value gradually decreased to 22.2 Å until 200 ns. Overall, these findings demonstrate how drastically the mutation affects each system’s internal dynamics.

### 3.5. Functional Displacement of GLA and Mutants

**Figure 5:**
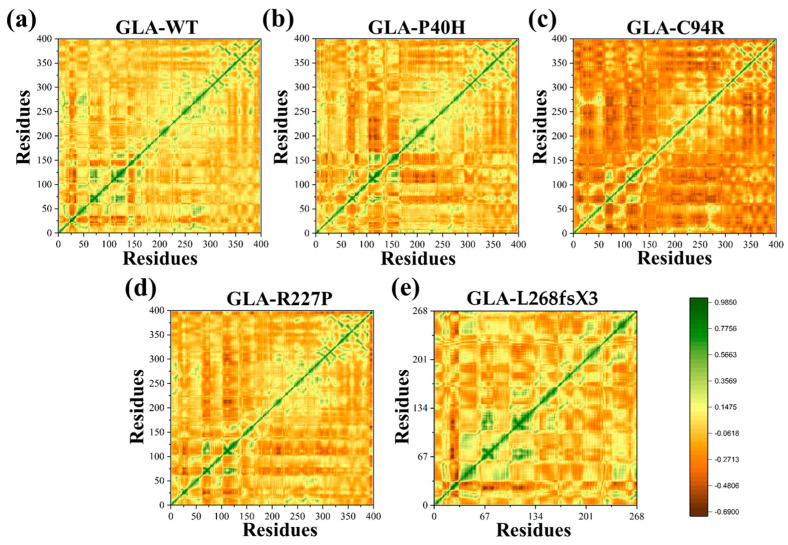
DCCM of the GLA wild type and mutants. (**a**) Wild type (**b**) GLA-P40H, (**c**) GLA-C94R, (**d**) GLA-R227P, and (**e**) GLA-L268fsX3.

The temporal functional displacement of both native GLA and its mutant variants over time was evaluated using the dynamic cross-correlation matrix (DCCM) approach. The results demonstrated a positive correlation between the C- and N-termini. In comparison to GLA (WT), a decrease in the positive association among the nearby residues at the mutation site was detected for the GLA-P40H variant. For the GLA-C94R variant, a pronounced manifestation of robust anticorrelated motion was observed extending beyond the mutation site to involve distinct residues at both ends, validating that the observed negative correlation may be attributed to the adopted molecular conformation. Furthermore, for additional missense variants of GLA, such as GLA-R227P and GLA-L268fsX3, the results demonstrated a strong positive correlation in comparison with the other missense variants, as shown in Figure 5. Overall, the findings of the DCCM analysis demonstrated that the native protein and mutant variants exhibited different patterns of significant positive and negative associations. A deep green color indicates stronger associations among residues, while a deep brown color indicates strong anticorrelation among the residues.

### 3.6. Impacts on the Internal Dynamics and Structures of GLA Mutants

**Figure 6:**
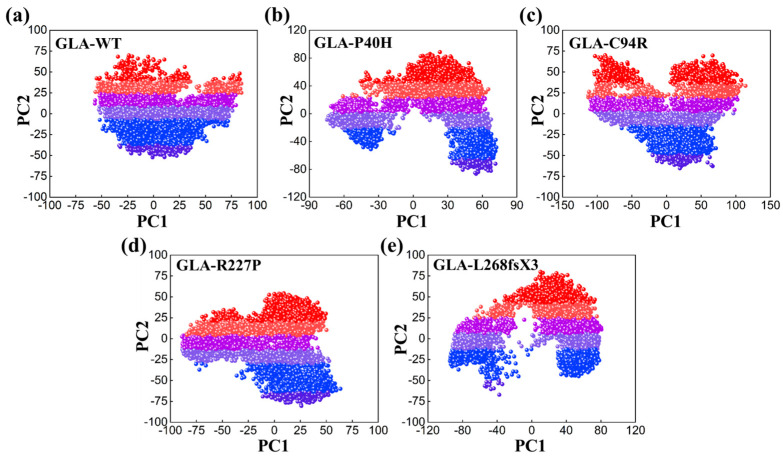
PCA plot of the GLA wild type and mutants, (**a**) wild type, (**b**) GLA-P40H, (**c**) GLA-C94R, (**d**) GLA-R227P, and (**e**) GLA-L268fsX3.

To study dynamic adaptive conformational alterations in the structure of the GLA wild type and mutants (GLA-P40H, GLA-C94R, GLA-R227P, and GLA-L268fsX3), principal component analysis (PCA) was applied. PCA is a statistical method that combines multiple correlated variables with a smaller number of uncorrelated variables termed principal components. We visualized the first two eigenvectors that contribute to the total motion movements associated with the underlying system. As shown in Figure 6, each dot represents a single confirmation. GLA-P40H, GLA-C94R, and GLA-L268fsX3 exhibited a unique pattern characterized by periodic jumps, indicating distinctive behavior. The native GLA and GLA-R227P strains exhibit compact movements. All these results indicate that owing to the influence of mutations, the internal dynamics of native GLA and its mutant variant systems have undergone considerable variation.

### 3.7. Impaired Enzyme Activities of GLA Mutants

**Figure 7:**
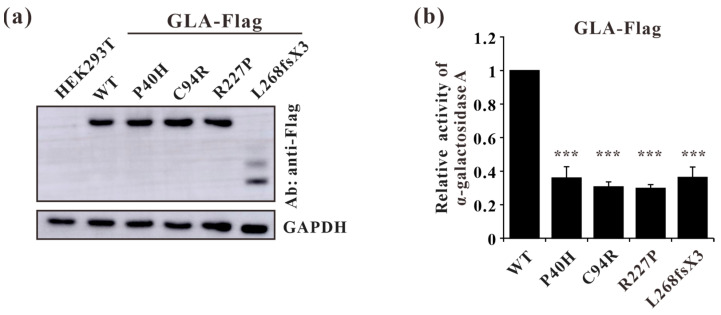
Validation of stable cell line constructions and α-galactosidase A-enzyme activity analysis of GLA wild type and mutants. (**a**) Western blot analysis of GLA-WT, P40H (c.119C>A), C94R (c.280T>C), R227P (c.680G>C), and L268fsX3 (c.801+1G>A) mutant stable overexpression cells; (**b**) A-galactosidase enzyme activity analysis of the GLA wildtype and mutants. Significant difference: *** *p* < 0.001.

To further characterize these four *GLA* mutations (c.119C>A, p.P40H; c.280T>C, p.C94R; c.680G>C, p.R227P; c.801+1G>A, p.L268IfsX3) at the cellular level, lentivirus plasmids containing *GLA* wildtype or mutants cDNA were constructed and the resulting mutations were verified by Sanger sequencing. The stable overexpression of C terminus Flag-tagged GLA wild-type or mutants in HEK293T cells were established and verified by Western blot (Figure 7a). Before comparing the α-galactosidase A enzyme activities between Flag-tagged GLA wild-type and mutants, the activity of untransfected HEK293T cells was subtracted as endogenous enzyme activity according to the manufacturer’s guidelines. The α-galactosidase A enzyme activities of all the GLA mutant-overexpressing cells (P40H, C94R, R227P, and L268fsX3) were significantly lower than that of the GLA wildtype-overexpressing cells, with a relative GLA enzyme activity down to 30% (Figure 7b). These results indicated that these mutations indeed affected the enzyme activity of α-galactosidase A.

### 3.8. GLA Mutants Cause Intracellular Accumulation of Autophagosomes

**Figure 8:**
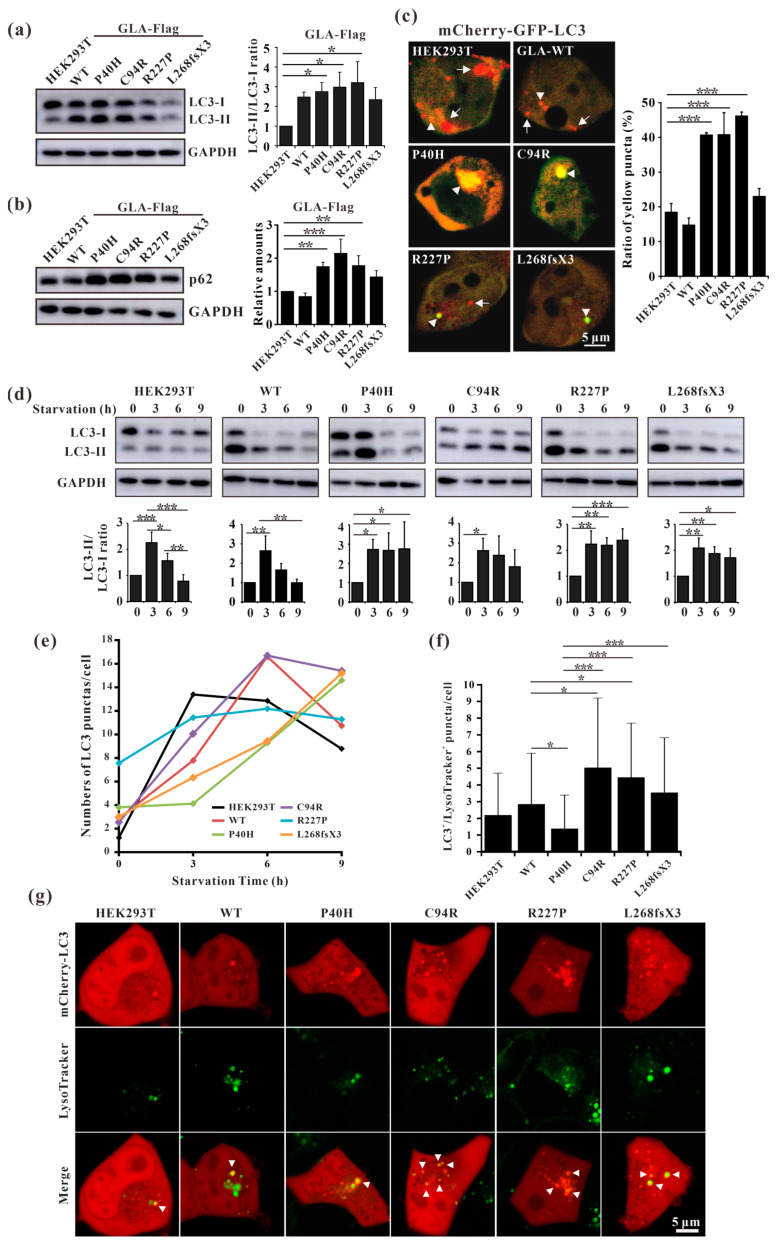
Overexpression of the GLA mutants impaired autophagy. (**a**) Western blot analysis of LC3-II expression in GLA wildtype and mutants’ overexpression cells; (**b**) Western blot analysis of p62 expression in GLA wildtype and mutants’ overexpression cells; (**c**) Immunofluorescence assay of pCS2-mCherry-GFP-LC3 transfection into the GLA wildtype and mutants’ overexpression cells, respectively; (**d**) Rario of LC3-II/LC3-I were analyzed upon starvation of indicated times; (**e**) LC3 puncta were quantified in each cell line upon starvation of indicated times; (**f**) The colocalization of LC3 and lysosome were quantified; (**g**) Immunofluorescence of each cell line that transiently infected with mCherry-LC3 and incubated with LysoTracker. Arrowhead indicates the colocalization of LC3 and LysoTracker. Significant difference: * *p* < 0.05, ** *p* < 0.01, *** *p* < 0.001.

Fabry disease is caused by the accumulation of the lipid substrate GB3 in cells due to the loss or decrease in α-galactosidase A activity, which leads to morphological changes and functional impairment of cells [34]. Autophagy is dysregulated in GLA-deficient podocytes and the autophagy pathway is impaired in Fabry patients [35,36]. Our results showed that the ratio of LC3-II/LC3-I and the expression level of p62 were both significantly increased in all the mutant-overexpressing HEK293T cells (Figure 8a,b), except for GLA-L268fsX3-overexpressing cells. Moreover, an immunofluorescence assay revealed that the number of yellow puncta indicating autophagosomes was significantly greater in all GLA-mutant-overexpressing cells after transfection with the pCS2-mCherry-GFP-LC3 plasmid (Figure 8c). To further verify whether the increase in autophagosomes resulted from accelerated formation or impaired autophagic flux, these cells were subjected to starvation treatment. Cell lysates from 0 h, 3 h, 6 h, and 9 h of starvation treatment were collected according to the time gradient, and Western blot analysis was performed. The results showed that the ratio of LC3-II/LC3-I was significantly increased after starvation for 3 h in all these cell lines, suggesting the induction of autophagosome formation upon starvation. Furthermore, the ratio of LC3-II/LC3-I decreased to normal levels with the extension of starvation time in HEK293T cells and in GLA-WT stable overexpression cells. However, the ratio of LC3-II/LC3-I did not significantly decrease after 9 h of starvation treatment in the cells stably overexpressing GLA (Figure 8d). Immunofluorescence results also showed that the puncta formation of mCherry-LC3 was increased after starvation for 3 h or 6 h in all cell lines and decreased after starvation for 9 h in HEK293T cells and in GLA-Flag-WT overexpressing cells. In the GLA mutants’ overexpressing cells, the number of LC3 puncta did not decrease (Figure 8e), suggesting the accumulation of autophagosomes in these cells. Autophagy is a very fast process, and the cargoes engulfed inside the autophagosome are degraded after fusion with the lysosome. Immunofluorescence revealed that the colocalization of mCherry-LC3 with the lysosome tracker was significantly increased in GLA-Flag-C94R- and GLA-Flag-R227P-overexpressing cells than in GLA-Flag-WT-overexpressing cells (Figure 8f,g), indicating that autophagic flux was impaired in these two cell lines. Taken together, our results suggested that the overexpression of these GLA mutants in HEK293T cells inhibited autophagy.

### 3.9. GLA Mutation Leads to an Increase of Lysosome Numbers in Cells

**Figure 9:**
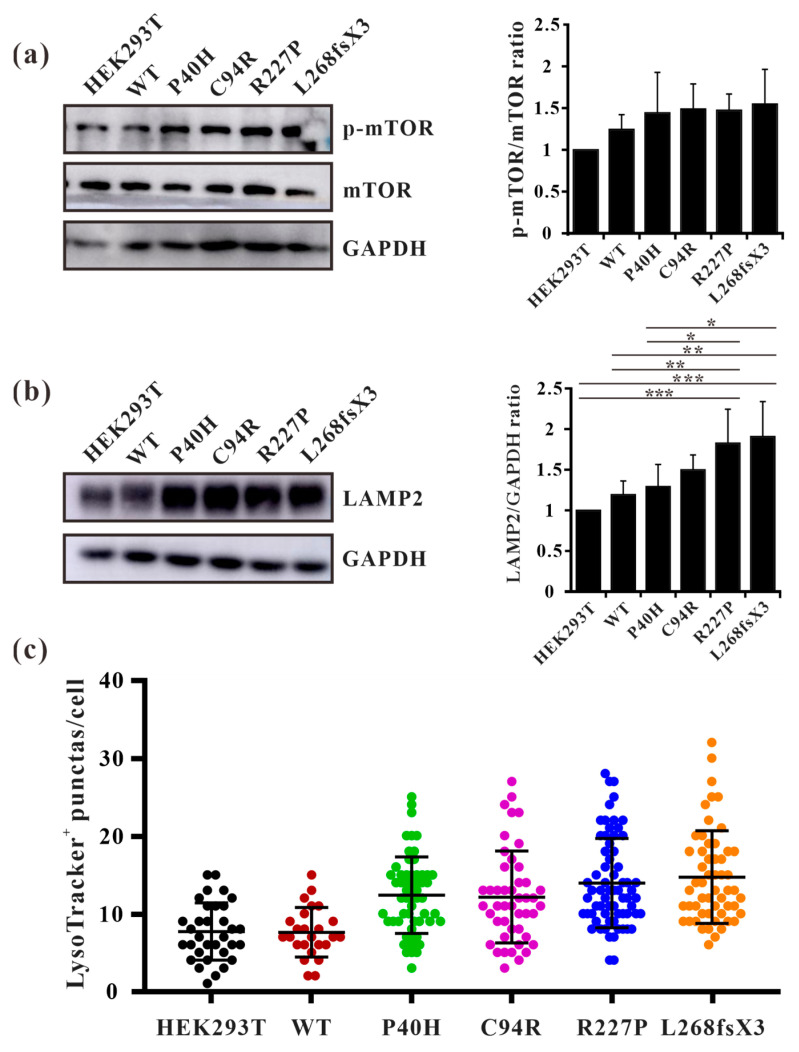
The numbers of lysosomes were increased in GLA mutants overexpressing cells. (**a**) Western blot analysis of mTOR phosphorylation; (**b**) Western blot analysis of LAMP2. (**c**) LysoTracker-positive puncta were quantified in each cell line. Significant difference: * *p* < 0.05, ** *p* < 0.01, *** *p* < 0.001.

Autophagy is regulated by upstream mTOR, which is a negative regulatory protein of autophagy and its phosphorylation inhibits autophagy [14]. The excessive occurrence of autophagy activates mTOR and thus negatively regulates autophagy [37]. We found that autophagosomes accumulate substantially in cells overexpressing GLA mutants, but it is unclear whether this accumulation affects intracellular mTOR expression levels. Western blot analysis also revealed that the phosphorylation level of mTOR in GLA mutants’ overexpressing cells was slightly increased (Figure 9a). This finding suggested that there may be a negative feedback regulatory mechanism in the cell; that is, the accumulation of autophagosomes in the cell activates mTOR and inhibits the excessive production of autophagosomes. It has been reported that the number of lysosomes is regulated by mTOR and that phosphorylation of mTOR can promote lysosome regeneration and lysosomal-related protein expression [38]. Furthermore, whether GLA, an enzyme located within lysosomes, causes damage to lysosomes is unknown. We detected the lysosomal marker LAMP2 via Western blotting, and the results showed that LAMP2 was slightly increased in Flag-GLA-P40H- and Flag-GLA-C94R-overexpressing cells, while LAMP2 was significantly increased in Flag-GLA-R227P- and Flag-L268fxX3-overexpressing cells (Figure 9b). Immunofluorescence also revealed that the number of LysoTracker-positive puncta was significantly elevated in GLA mutants’ overexpressing cells (Figure 9c). These results suggest that the overexpression of the GLA mutants leads to an increase in the number of lysosomes.

## 4. Discussion

In this study, we analyzed the effects of four *GLA* mutations on the structure of α-galactosidase A (Figure 1, Figure 2, Figure 3, Figure 4, Figure 5 and Figure 6). *In silico* analysis revealed that all four of these mutations impair the structure of the protein and therefore might destroy its stability (Figure 1, Figure 2, Figure 3, Figure 4, Figure 5 and Figure 6). These mutations resulted in a significant decrease in α-galactosidase A activity (Figure 7). A previous study showed that autophagy was dysregulated in GLA-deficient podocytes [36], and our results also indicated that the overexpression of GLA mutants inhibited autophagy (Figure 7 and Figure 8). Compared with those in HEK293T cells, the ratio of LC3-II/LC3-I and the expression level of p62 in GLA-L268fsX3-overexpressing cells were slightly increased. This difference might be due to the instability of the L268fsX3 protein since the expression level of L268fsX3 is much lower than that of the other mutants (Figure 7a). Furthermore, we found that in control and GLA-WT overexpressing cells, starvation significantly induced autophagy, but with prolonged starvation, cell degradation accelerated. Therefore, after 9 h of starvation, the expression of LC3-II in the cells returned to normal. However, in the cells overexpressing these four GLA mutants, the expression of LC3-II did not significantly decrease after 9 h of starvation treatment, suggesting that autophagy was induced in these cells during starvation treatment. However, due to the disruption of lysosomal substrate degradation, autophagosomes cannot be rapidly degraded and accumulate in cells (Figure 8). These results indicate that upon *GLA* mutation, the cellular autophagy flux is blocked and autophagy is disrupted which is consistent with the previous results from FD patients’ cells and *GLA* knockdown/knockout cell lines [36,39,40].

The initiation of autophagy is regulated by upstream mTOR, and the activity of mTOR is also regulated by upstream kinases, such as AMPK and AKT. Multiple phosphorylation sites of mTOR have been found, among which S2448 can be phosphorylated by upstream of AKT or S6K1 kinase to enhance the activity of mTOR [41]. Under adequate nutrition conditions, mTOR inhibits the occurrence of autophagy by phosphorylating related downstream proteins, while under starvation conditions, autophagy is activated through the inhibition of mTOR [42]. Several studies have demonstrated that while regulating autophagy, mTOR is also subject to feedback regulation during autophagy [37]. In podocytes, autophagy was impaired and the activity of mTOR was decreased upon *GLA* knockdown. The authors suggested that the decreased phosphorylation of mTOR led to the increase of autophagosomes [36]. However, long-term activation of autophagy also results in damage to cells. Our study showed that the *GLA* mutations cause the accumulation of autophagosomes in GLA mutants’ overexpression cells, but the activity of mTOR seems to be enhanced (Figure 9a). Therefore, we speculate that there should be a negative feedback regulation mechanism that the increased autophagosomes could activate mTOR to inhibit autophagosome formation and stimulate lysosome regeneration. This regulatory mechanism probably protects cells from death due to excessive autophagy.

After autophagosome formation, the lysosomes are fused to form phagolysosomes, which degrade their inclusions for cell reuse. However, the effects of *GLA* mutations on lysosomes remain unknown. Lossing of α-galactosidase A activity causes the accumulation of Gb3 in the lysosome of FD patients. Therefore, the function of the lysosome might be affected by the *GLA* mutation. Further, studies have shown that the expression levels of LAMP1 and LAMP2 are increased in FD patients [43]. Our results also showed significantly increased expression levels of LAMP2 in GLA mutants’ overexpressing cells (Figure 9b), suggesting that *GLA* mutations resulted in an increased number of intracellular lysosomes. Studies have shown that the number of lysosomes is regulated by mTOR, and the activation of mTOR can promote the expression of lysosome-related proteins. Although the inhibition of mTOR can induce the generation of autophagosomes, it also inhibits the regeneration of lysosomes, which exhibit strong degradation ability [38]. In GLA mutants’ overexpressing cells, autophagy flux was blocked, autophagosomes accumulated in large amounts, and the degradation ability of lysosomes was decreased. The intracellular negative feedback regulation increased the phosphorylation of mTOR, which in turn inhibited the continuous production of autophagosomes and promoted the regeneration of lysosomes, as well as the expression of lysosome-related proteins, thereby increasing the number and activity of lysosomes which might accelerate the degradation of harmful substances or damaged organelles, and ultimately promote cell survival.

Our study demonstrated that these four *GLA* mutations resulted in a decrease of α-galactosidase A activity and impaired autophagy. The intracellular negative feedback regulation increases the activity of mTOR, which not only inhibits the occurrence of excessive autophagy in cells but also enhances lysosome degradation and promotes cell survival. Precision medicine refers to medical adaptation according to the individual characteristics of each patient [44]. It is based on the ability to classify individuals into subgroups reflecting different risks. For example, compared to patients with missense mutations, patients with nonsense mutations often exhibit severe disease phenotypes which are recommended more frequently to initiate therapy. Moreover, chaperone therapy is only suitable for patients with an amenable mutation, and enzyme replacement therapy (ERT) is required for all others. Further, early initiation of therapy is particularly needed in men with significantly reduced GLA activity, elevated lysosomal Gb3, and organ manifestations typical of FD. Especially, women carrying the *GLA* p.N215S mutations (late-onset mutations) may have not received treatment for many years, saving costs and avoiding side effects. Finally, patients with “benign” *GLA* variants should not be treated with FD-specific therapies [44]. Therefore, our results from these four *GLA* mutations will deepen our understanding of the pathogenic mechanism of FD and broaden our insight into the clinical treatment of FD.

## Data Availability

All primary data are available upon reasonable request.
